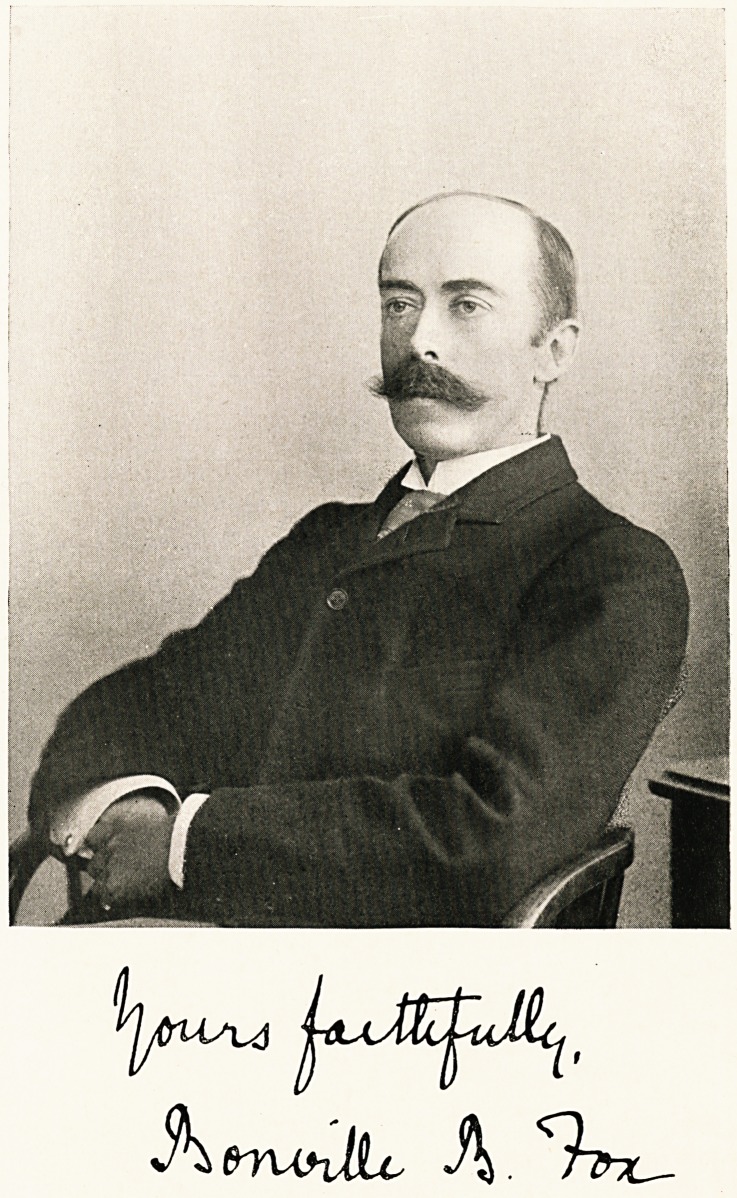# Bonville Bradley Fox

**Published:** 1902-06

**Authors:** 


### BONVILLE BRADLEY FOX, M.A., M.D. (Oxon.)

WE record with sincere regret the death of Dr. Bonville Bradley Fox, of Brislington House, at the early age of 49, a few days only after that of his half-brother, Dr. Edward Long Fox, of Clifton. Dr. Bonville Fox, like his brother, took a warm interest in the Bristol Medico-Chirurgical Society, and until his health failed was a regular attendant at its meetings, and a valued contributor to any discussion connected with nervous or mental diseases. He would indeed a few years ago have occupied the Presidential Chair, but reasons of health compelled him to decline this office.

Dr. Bonville Fox was the son of Dr. Francis Ker Fox by his second wife, the eldest daughter of the Rev. Charles Bradley, and sister of the present Dean of Westminster, and of the wife of Dr. E. Long Fox. He was educated at Dr. Hudson's School, Manilla Hall, Clifton, and at Marlborough College, subsequently proceeding to Christ Church, Oxford, whence he graduated M.A. in 1870. The medical part of his education he received at St. George's Hospital. After taking the M.R.C.S. in 1878, he acted for six months as Assistant Resident Medical Officer at Bethlehem Hospital. He took his M.B. Oxford in 1879, and M.D. in 1882. After leaving Bethlehem he became Resident Medical Officer at Brislington House, which, under the administration of his father, had become one of the best known asylums in the country, and has ever since maintained its reputation.

On the death of his father he became joint proprietor with his brother, Dr. Charles Fox, and sole proprietor since the retirement of the latter.

Dr. Bonville Fox married the daughter of the late Mr Tom Danger, who was for many years Clerk of the Peace for this city, and leaves a family of two sons and one daughter.

He was widely known, and held a high position in his own branch of medicine, and his advice in cases belonging to his speciality was frequently sought and highly valued, being the fruits of an extensive experience and sound judgment. He contributed the article on "Exaltation" to Tuke's *Dictionary of Psychological Medicine*, several papers to the *Journal of Mental Science*, and one to this *Journal* on a very remarkable case of "hystero-epilepsy." He was an excellent and thoughtful speaker, and his contributions to the debates at the local medical societies were invariably listened to with respect and attention.

In addition to his heavy professional work, Dr. Bonville Fox found time to devote to public matters. He was for many years a member of the Keynsham Board of Guardians, where he showed himself to possess great business abilities, and was for a considerable period vice-chairman. He was a strong Conservative in politics, and an active worker in the cause of his party, rendering great assistance to the Brislington District Conservative Committee. He was also strongly interested in agricultural matters, and was a useful member of the committee of the North-East Somerset Farmers' Club. Dr. Fox was fond of all outdoor pursuits, was a good shot, and an enthusiastic lover of cricket. He himself was a cricketer of more than average merit, and was a valued member of the village club, which he frequently captained. In private life Dr. Bonville Fox was a most witty and genial companion, well-informed and widely read: to those who had the privilege of his friendship he was a warm and constant friend. He was greatly esteemed by his professional brethren, and by a large circle of friends and neighbours. His death followed on a long and painful illness, and the sufferings it involved were borne by him with patience and fortitude. He was buried in the private cemetery of the family at Brislington on April 5th, when a large company assembled, the ladies and gentlemen of Brislington House and the staff being well represented, together with many relatives and friends from a distance.

Dr. Lionel A. Weatherly writes of him :—

"Dr. Bonville Fox will be much missed by the members of the Medico-Psychological Association. For years he was a very regular attendant at the quarterly meetings, and he had been a member of the Council. His thoughtful and scholarly mind was appreciated by all who knew him, and whenever he rose to speak he commanded immediate attention and respect, for all the members of the Association recognised that he seldom brought forward an opinion which had not been carefully weighed and logically reasoned. All remember his capital paper on 'Exaltation in Chronic Alcoholism,' and the interesting discussion which followed, while those of the Association who were so fortunate as to be his guests at the meeting held at Brislington House, on May 1st, 1891, will long remember that pleasant day and his courteous and kind hospitality. His death causes a gap in the Association not easily to be filled, and we mourn with many the loss of a kind heart, a scholarly mind, and an ever-thoughtful courtesy."

We are glad to be informed that Brislington House Asylum will be conducted as heretofore, for Mrs. Bonville Fox will continue to take an active interest in the welfare of the inmates in conjunction with the two medical officers.

---

### BIBLIOGRAPHY OF THE WORK OF BONVILLE BRADLEY FOX.

"Case of Acute Dementia of Rapidly Fatal Termination," *J. Ment. Sc.*, 1882, xxvii. 212.

"Exaltation in Chronic Alcoholism," *Ibid.*, 1885, xxx. 233, 644.

"A Case of Masked Epilepsy," *Bristol M.-Chir. J.*, 1886, iv. 172.

"Some Unusual Cases of General Paralysis," *J. Ment. Sc.*, 1891, xxxvii., 389

Article on "Exaltation" in *A Dictionary of Psychological Medicine*, Edited by D. Hack Tuke, vol. i., 1892.

"A Case of Hystero-Epilepsy," *Bristol M.-Chir. J.*, 1896, xiv. 224.

"A Medico-Legal Case," *J. Ment. Sc.*, 1896, xlii. 114.

## Figures and Tables

**Figure f1:**